# LAMP-based foldable microdevice platform for the rapid detection of *Magnaporthe oryzae* and *Sarocladium oryzae* in rice seed

**DOI:** 10.1038/s41598-020-80644-z

**Published:** 2021-01-08

**Authors:** M. K. Prasannakumar, P. Buela Parivallal, Devanna Pramesh, H. B. Mahesh, Edwin Raj

**Affiliations:** 1 Department of Plant Pathology, University of Agricultural Sciences, Bangalore, India; 2 Rice Pathology Laboratory, All India Coordinated Rice Improvement Programme, University of Agricultural Sciences, Raichur, India; 3 Department of Genetics and Plant Breeding, College of Agriculture, V.C. Farm, Mandya, India; 4 Plant Pathology Division, ICAR - National Research Center for Banana, Thayanur, India

**Keywords:** Microbiology, Plant sciences

## Abstract

Rice blast (caused by *Magnaporthe oryzae*) and sheath rot diseases (caused by *Sarocladium oryzae*) are the most predominant seed-borne pathogens of rice. The detection of both pathogens in rice seed is essential to avoid production losses. In the present study, a microdevice platform was designed, which works on the principles of loop-mediated isothermal amplification (LAMP) to detect *M. oryzae* and *S. oryzae* in rice seeds. Initially, a LAMP, polymerase chain reaction (PCR), quantitative PCR (qPCR), and helicase dependent amplification (HDA) assays were developed with primers, specifically targeting *M. oryzae* and *S. oryzae* genome*.* The LAMP assay was highly efficient and could detect the presence of *M. oryzae* and *S. oryzae* genome at a concentration down to 100 fg within 20 min at 60 °C. Further, the sensitivity of the LAMP, HDA, PCR, and qPCR assays were compared wherein; the LAMP assay was highly sensitive up to 100 fg of template DNA. Using the optimized LAMP assay conditions, a portable foldable microdevice platform was developed to detect *M. oryzae* and *S. oryzae* in rice seeds. The foldable microdevice assay was similar to that of conventional LAMP assay with respect to its sensitivity (up to 100 fg), rapidity (30 min), and specificity. This platform could serve as a prototype for developing on-field diagnostic kits to be used at the point of care centers for the rapid diagnosis of *M. oryzae* and *S. oryzae* in rice seeds. This is the first study to report a LAMP-based foldable microdevice platform to detect any plant pathogens.

## Introduction

Rice (*Oryza sativa* L.) is an important food grain globally^1^. More than 90% of rice is being grown in China, Japan, India, Pakistan, Vietnam, and Thailand^2^. Rice production is affected by the various biotic and abiotic constraints, which reduce grain yield and quality. Out of 52 fungal pathogens infecting rice, forty-one reported being seed-borne^3,4^, among them, *Magnaporthe oryzae* and *Sarocladium oryzae* are responsible for causing destructive diseases such as blast and sheath rot, respectively^5,6^. *M. oryzae* was reported to be present on the seed or in the tissues of embryo, endosperm, bran layers, and glumes, whereas *S. oryzae* has been isolated from the husks and kernels of rice seeds^5^. Seed-borne plant pathogens cause yield and quality losses, as well as a vital source of the pathogen, spread, and dissemination^7^. The number of seed-borne pathogens that threaten rice production is exceptionally high, and, therefore, the maintenance of high-quality rice seed is necessary to secure food production^3,6^. Early diagnosis of seed-borne fungal pathogens is essential to avoid uncontrolled propagation of pathogens through the long-distance exchange of such material, which prevents economic losses and unnecessary use of fungicides^8^.

Several conventional methods, viz., visual observation, and incubation techniques, can detect seed-borne fungi in rice^3,5^. Although conventional techniques are used frequently due to their simplicity, they are less sensitive, time-consuming, require mycological skills, and challenging to apply for a large number of samples^8,9^. Recently, highly sensitive, robust, and pathogen-specific techniques based on nucleic acid (NA) have been developed^6,8,9^. The most common method is conventional polymerase chain reaction (PCR)^10^, while other recent methods include nested PCR^11^, multiplex PCR^6^, real-time PCR^9,11^and Bio-PCR^12^ have been developed for detecting seed-borne fungi in rice and vegetables. Even though NA-based techniques are highly sensitive and specific, which encounter many problems like the low quality of template DNA due to the presence of PCR inhibitors in seeds during the assay^8^. In addition to the above, the conventional PCR techniques also have other drawbacks such as the requirement of sophisticated instruments like thermocycler and gel documentation systems. To address these disadvantages, several isothermal amplification methods, such as loop-mediated isothermal amplification (LAMP)^13^, helicase dependant amplification (HDA)^14^, nucleic acid sequence-based amplification^15^, etc., have been developed^16^.

The LAMP assay has been widely used to detect viruses^17^, bacteria^18,19^, and fungal pathogens^20^ due to its high sensitivity and specificity compared to other conventional PCR methods^19^. The HDA assay involves in vivo DNA replication using *helicase* enzyme^14,16,21,22^ and does not require initial heat denaturation and subsequent thermocycling steps as required by PCR. Based on the principles of the LAMP assay, several microdevices have been developed integrating foldable technology^23–25^. A foldable LAMP microdevice for fuchsin-based colorimetric detection of multiple food-borne pathogens has been developed by^24^, where a foldable membrane fully integrating DNA purification, amplification, and detection processes for detecting multiple food-borne pathogens has been standardized. Although the LAMP and other isothermal assays have been developed to detect several plant pathogens, the foldable microdevices are not available for any plant pathogen. Foldable microdevice can be effectively utilized at the point-of-care centers for the rapid diagnosis of the plant pathogen without using any sophisticated instruments.

This study has developed two isothermal amplification techniques for the rapid, sensitive, specific, and cost-effective detection of *M. oryzae* and *S. oryzae* in the rice seeds. The relative sensitivity of isothermal amplification methods and PCR based techniques were compared. Further, a portable, foldable membrane-based microdevice platform has been developed using the basic fuchsin dye. It can be effectively utilized for performing the LAMP assay in the place of polypropylene tubes. The membrane microdevice platform designed in this study can be a prototype for developing point-of-care diagnostic kits used by the field extension service persons.

## Results

### PCR and qPCR assay

The PCR and qPCR assay for the *M. oryzae* and *S. oryzae* using pathogen-specific primers showed positive results when the respective pathogen's DNA template was used. Similarly, both assays detected both *M. oryzae* and *S. oryzae* when template DNA from the infected rice seeds was used. No amplification was detected when template DNA from the healthy rice seeds was used. The PCR and qPCR primers of *M. oryzae* and *S. oryzae* yielded negative results when DNA template of *Bipolaris oryzae, Rhizopus oryzae, Aspergillus flavus, Cladosporium fulvum,* and *Penicillium sp*. was used, which confirmed the pathogen-specificity of the primers and the assays. Further, the PCR amplification was confirmed on a 1.5% agarose gel.

### The HDA assay

For the HDA assay, the thermal stability was determined by incubating the *helicase* at 65 °C at different times. The assay for the ATPase activity was carried out with substrates at the experimentally determined optimal temperature of 55 °C for 10 min. The assay was carried out separately for *M. oryzae* and *S. oryzae* detection. The amplified products were analyzed on a 1% agarose gel where amplification was observed when DNA template from the fungal cultures (*M. oryzae* and *S. oryzae*) or infected seeds was used, whereas; no amplification was detected when DNA template of healthy seeds or *B. oryzae* was used. Similarly, HDA assay was carried out for DNA template of *B. oryzae, R. oryzae, A. flavus, C. fulvum,* and *Penicillium sp,* which resulted in the negative amplification, thus confirmed the primer and assay specificity.

### Optimization of the LAMP assay

**Figure 1:**
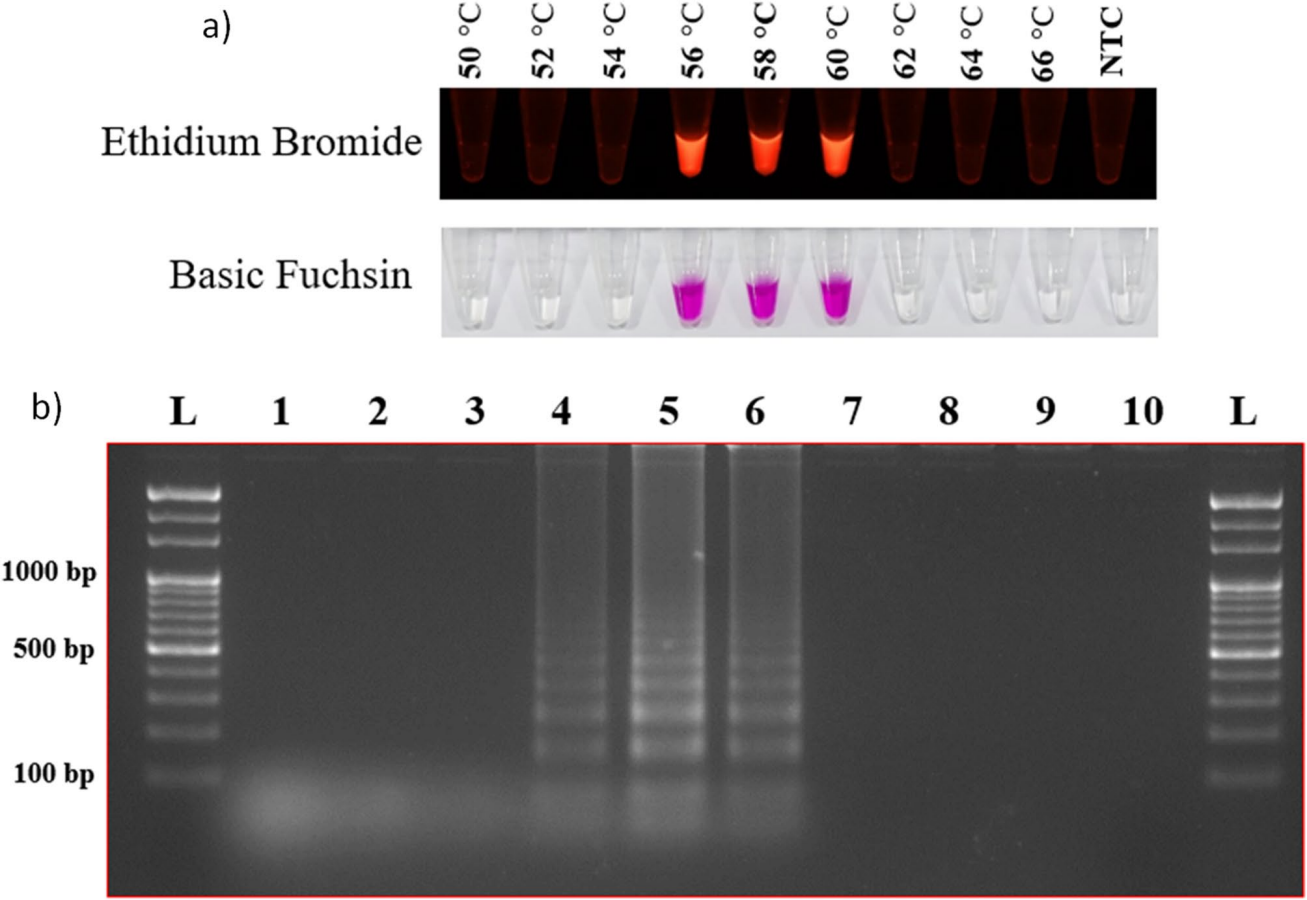
Reaction temperature optimization. (**a**) Temperature optimization (ranged between 50 to 66 °C) for the LAMP assay with Ethidium bromide and basic fuchsin dyes. (**b**) Further, results were visualized on a 2% agarose gel. Where lanes 1 to 9 indicate the temperature in °C (50, 52, 54, 56, 58, 60, 62, 64, and 66), lane N indicates negative control, and Lane L indicates 1 kb ladder (Thermo Scientific, USA).

**Figure 2:**
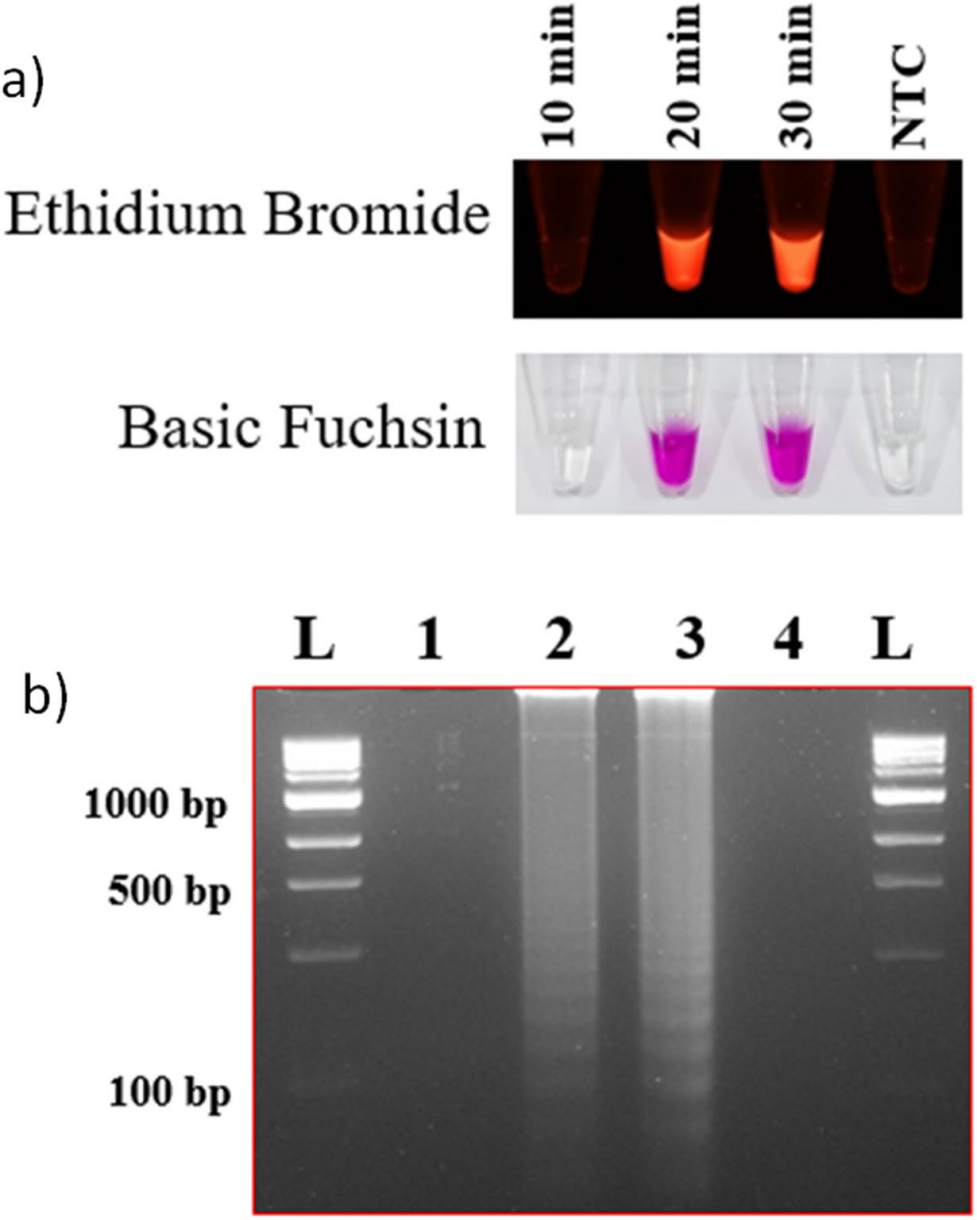
Reaction time optimization. Time optimization for the LAMP assay with Ethidium bromide and basic fuchsin (**a**) where NTC indicates non-template control. The results were visualized in 2% agarose gel (**b**), lane 1 to 3 indicates reaction time in min (10, 20, and 30), lane 4 indicates negative control, and the lane L indicates 1 kb ladder (Thermo Scientific, USA).

The reaction was optimized based on three key criteria, *i.e*., time, temperature, and target DNA template concentration. Template DNA of both *M. oryzae* and *S. oryzae* was used to optimize the LAMP assay. Colour differentiation between positive and negative results for detecting *M. oryzae* and *S. oryzae* was the primary criterion for selecting dyes. Both dyes (EtBr and basic fuchsin) used in the study were sensitive for the detection. EtBr showed fluorescence under UV light for positive amplification, whereas basic fuchsin showed purple color for a positive result and colorless for a negative result. To determine the optimum temperature and reaction time for the assay, a short-range of temperatures (56 °C to 60 °C) (Fig. 1) was used to analyze the highest fluorescence intensity and minimum time (15–90 min) (Fig. 2) using template DNA of the *M. oryzae* and *S. oryzae*. The assay was positive at different temperatures. The results were also assessed on a 2% agarose gel. No amplification was observed at 15 min incubation time, whereas positive results were observed after 30 min of incubation. Therefore, *M. oryzae* and *S. oryzae* can be best detected using either EtBr (under UV light) or basic fuchsin dyes under optimized conditions at 56 °C for 20 min.

### The specificity of the LAMP assay

**Figure 3:**
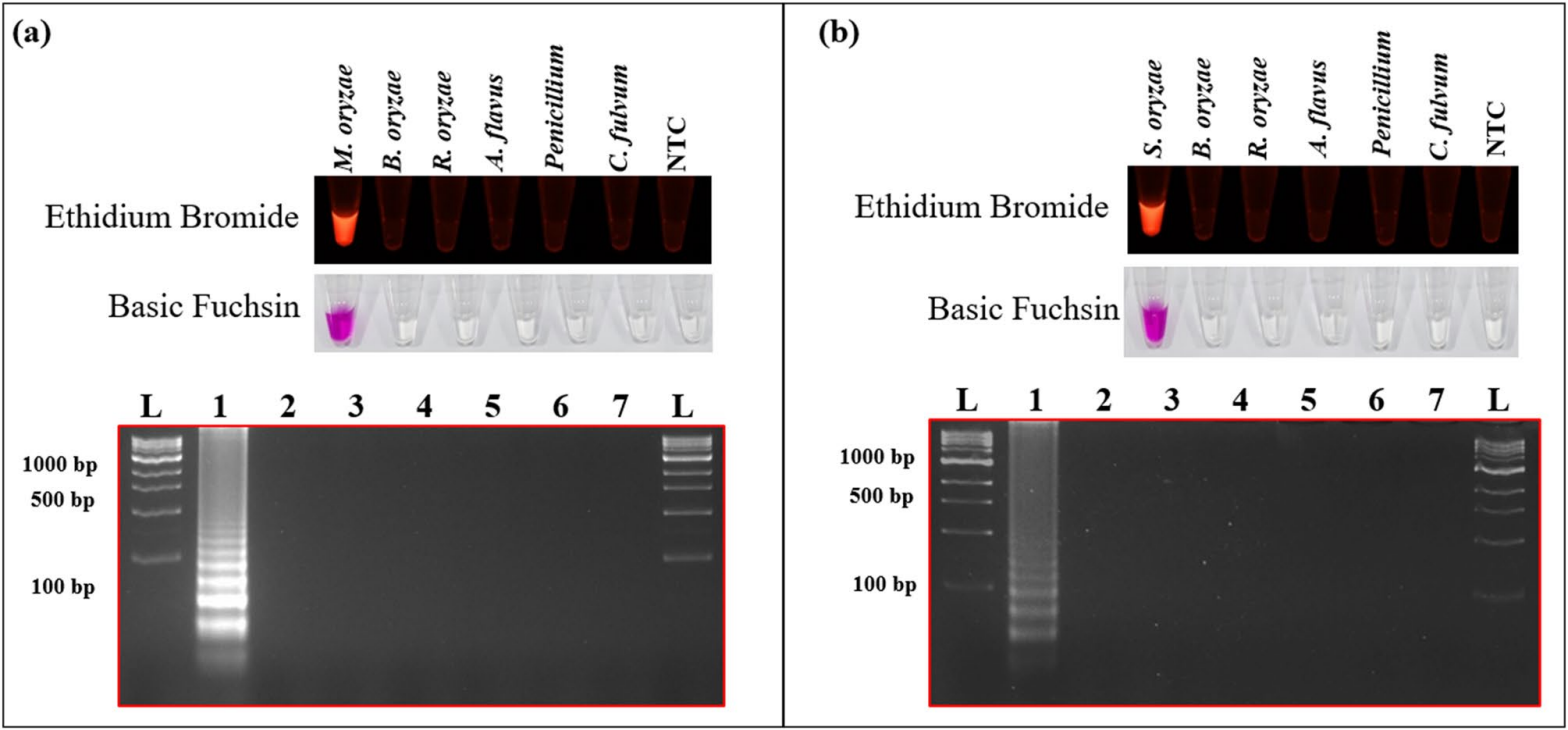
The specificity of LAMP assay. The specificity of the LAMP assay was validated for (**a**) *Magnaporthe oryzae* and (**b**) *Sarocladium oryzae* against *Bipolaris oryzae, Rhizopus oryzae, Aspergillus flavus, Cladosporium fulvum,* and *Penicillium* sp*.* using ethidium bromide and basic fuchsin. NTC indicates non-template control.

The LAMP assay's specificity for detecting *M. oryzae* and *S. oryzae* was evaluated using the template DNA of *B. oryzae, R. oryzae, A. flavus, Penicillium* sp. and *C. fulvum.* The template DNA of *M. oryzae* showed positive results when amplified in the LAMP assay using *M. oryzae* specific primers. Whereas the DNA from *S. oryzae, B. oryzae, R. oryzae, A. flavus, Penicillium* sp., and *C. fulvum* showed negative results, confirming the specificity of the LAMP assay (Supplementary Table 1 and Fig. 3a). Similarly, *S. oryzae* specific LAMP primers showed positive amplification only with the template DNA of *S. oryzae,* whereas; results were negative with template DNA of *M. oryzae* and *B. oryzae, R. oryzae, A. flavus, Penicillium,* and *C. fulvum* (Supplementary Table 1; Fig. 3b). All the LAMP assay result was reconfirmed by 2% agarose gel electrophoresis, and the absorbance intensity for the dye was also validated.

### The sensitivity of different nucleic acid amplification methods

**Figure 4:**
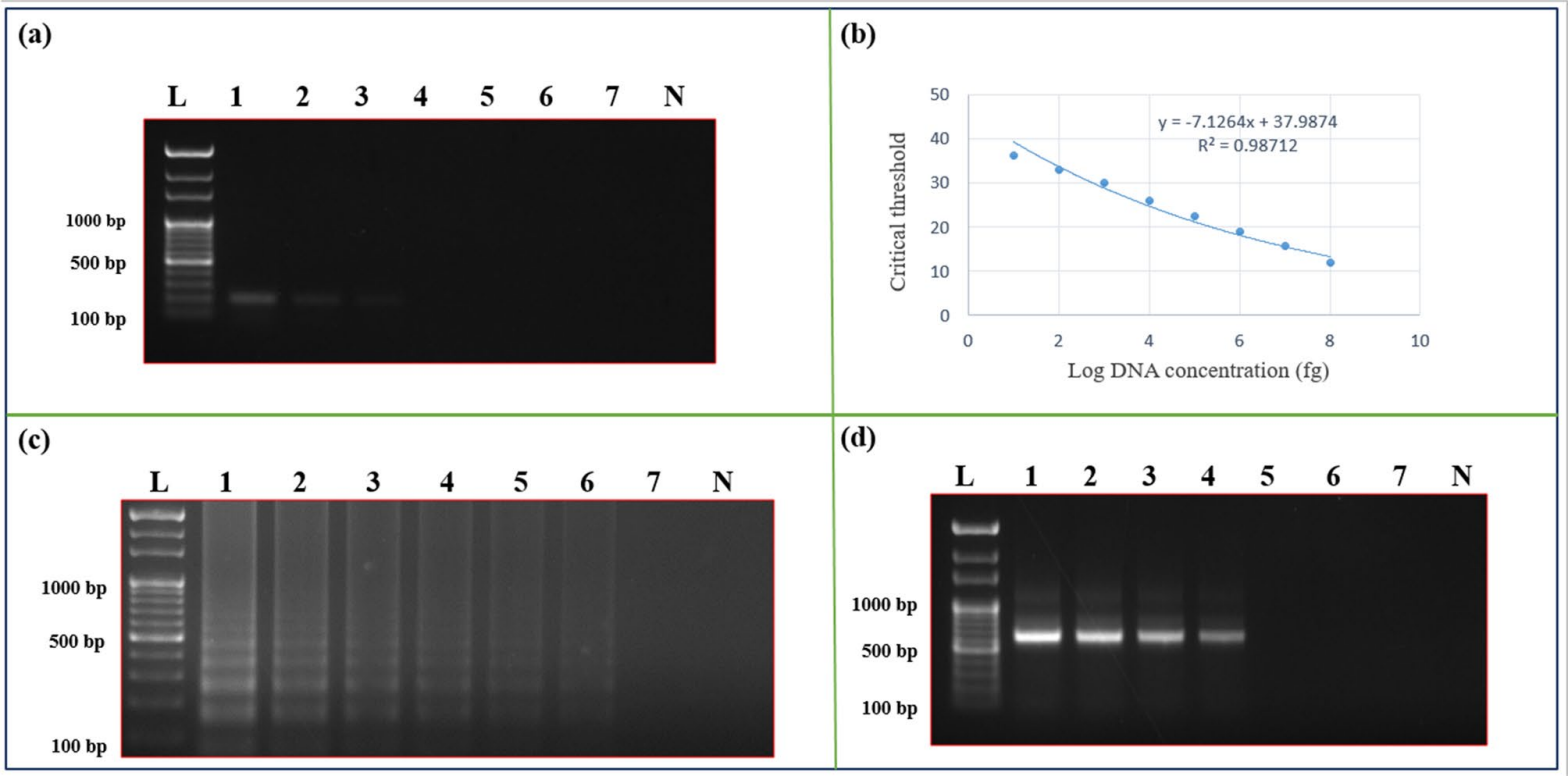
Comparative analysis of PCR, qPCR, LAMP assay, and HAD for their sensitivity using *Sarocladium oryzae* specific primers. (**a**) The sensitivity of the PCR assay, the result was visualized in a 1% agarose gel. Where lane L indicates 1 Kb ladder (Thermo Scientific), lanes 1–7 indicates 10 ng, 1 ng, 100 pg, 50 pg, 10 pg, 100 fg, 50 fg, and lane N indicates negative control. (**b**) The sensitivity of the qPCR assay. The standard curve represents log10 of DNA concentration in fg against Ct (cycle threshold) values for different DNA concentrations. (**c**) The sensitivity of the LAMP assay. The result was visualized in a 2% agarose gel. Lanes (1 to 7) indicates template DNA concentrations (10 ng, 1 ng, 100 pg, 50 pg, 10 pg, 100 fg, and 50 fg); lane N indicates negative control. (**d**) The sensitivity of the HDA assay. The result was visualized in a 1% agarose gel. Lanes (1 to 7) indicates template DNA concentrations (10 ng, 1 ng, 100 pg, 50 pg, 10 pg, 100 fg, and 50 fg), lane N indicates negative control, and lane L indicates 1 kb ladder (Thermo Scientific).

**Figure 5:**
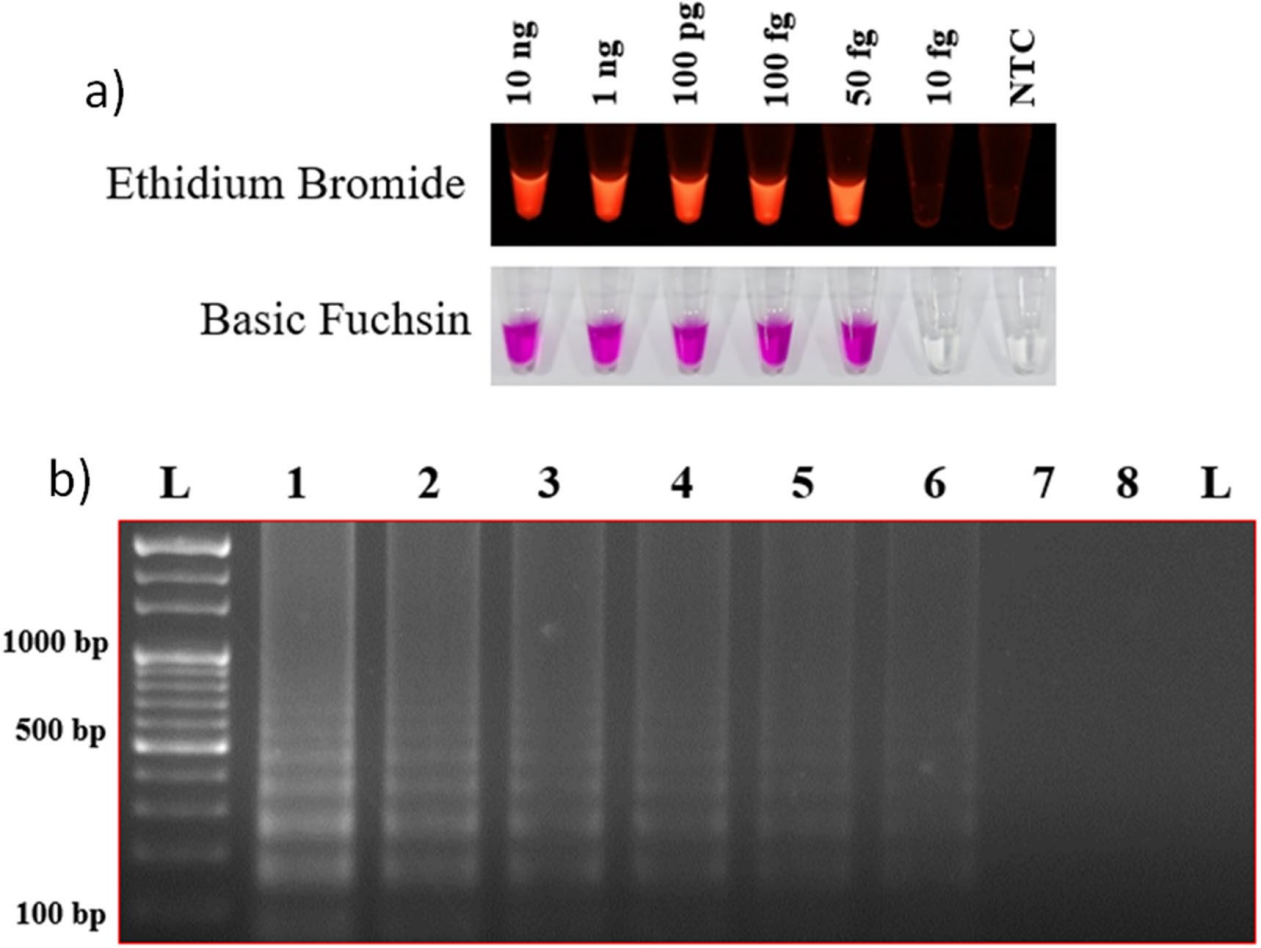
Reaction template-concentration optimization. Template-concentration optimization (10 ng to 50 fg) result was visualized using EtBr and basic fuchsin (**a**). Further, the result was confirmed on a 2% agarose gel (**b**), where the lanes (1 to 7) indicate template DNA concentrations (10 ng, 1 ng, 100 pg, 50 pg, 10 pg, 100 fg, and 50 fg), lane N indicates negative control, and the ane L indicates 1 kb ladder (Thermo Scientific).

The sensitivity of PCR methods (conventional PCR and qPCR) and isothermal amplification methods (the LAMP and the HDA assays) was analyzed using different DNA template concentrations ranging from 10 ng to 50 fg (of *M. oryzae* and *S. oryzae*). Conventional PCR showed positive results down to 1 ng, whereas the qPCR showed a positive result down to 10 pg. Among the isothermal assays, the HDA assay showed positive results down to 50 pg, whereas the LAMP assay showed positive down to 100 fg (Fig. 4). Therefore, the LAMP assay was the most sensitive among the different methods tested in our study for the positive detection of *M. oryzae* and *S. oryzae* infection in rice seeds (Fig. 5).

### Foldable membrane microdevice

**Figure 6:**
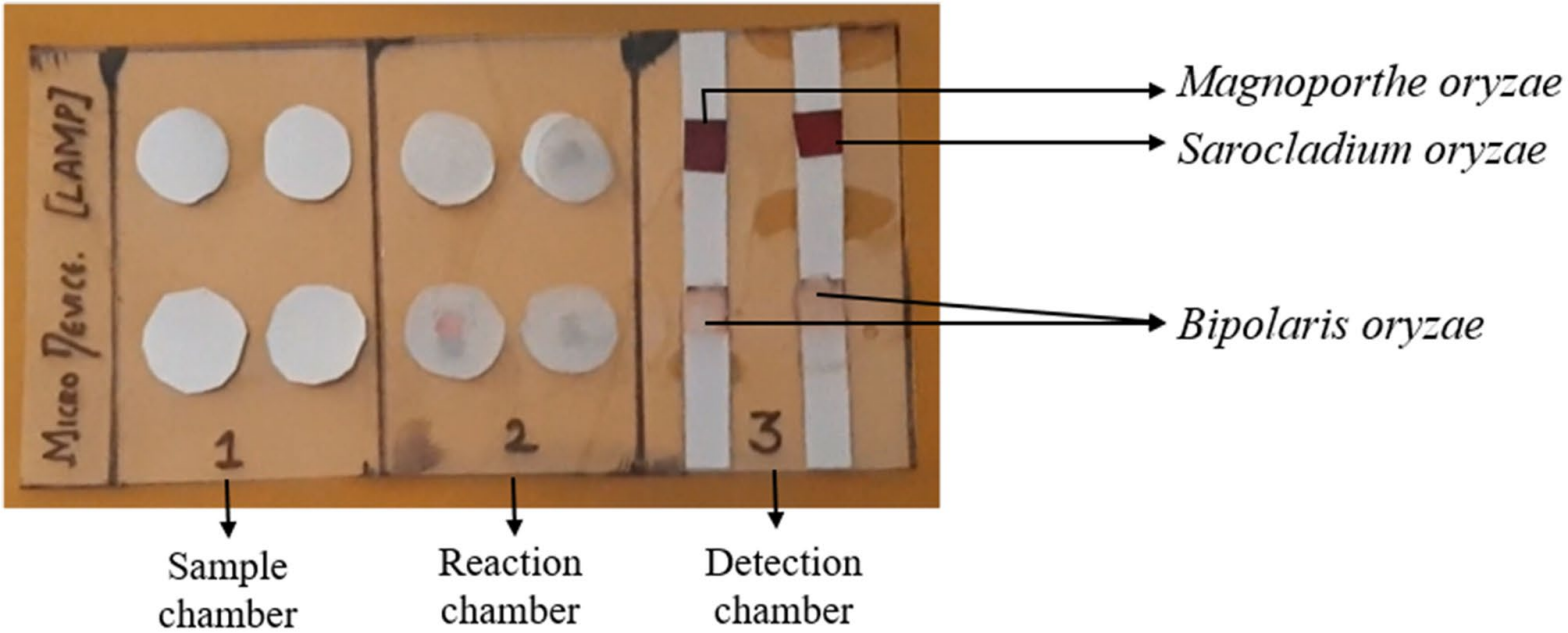
Detection of *Magnaporthe oryzae* and *Sarocladium oryzae* on foldable membrane microdevice platform. The result was visualized using basic fuchsin dye.

The LAMP assay for *M. oryzae* and *S. oryzae* detection was carried out on a newly developed foldable membrane-based microdevice. The assay was performed separately for *M. oryzae* and *S. oryzae,* and the result was observed at the detection chamber. The foldable microdevice detected *M. oryzae* and *S. oryzae* genome when the DNA template from the pure cultures or the infected seeds was loaded in the sample chamber, whereas no amplification was observed for the DNA template of healthy seeds. The fuchsin coated membrane at the detection chamber was turned into a purple color for the positive amplification, whereas it turns magenta to colorless for negative results (Fig. 6). In the foldable microdevice-LAMP assay, the pathogen-specific LAMP primers showed positive amplification only when the respective pathogen template DNA was present in the reaction chamber, whereas; negative results were observed when template DNA from either healthy seeds or nonspecific pathogen (*B. oryzae, R. oryzae, A. flavus, Penicillium* and *C. fulvum*) was added, indicating its specificity as that of the LAMP assay carried out in polypropylene tubes.

The assay was optimized for different incubation times, temperatures, and template concentrations. Like the LAMP assay, in the foldable microdevice, the genome of target pathogens can be detected at the optimized conditions of 56 °C for 20 min using a minimum of 100 fg of DNA template.

## Discussions

Among the forty-one seed-borne pathogens of rice, *M. oryzae* and *S. oryzae* are responsible for destructive diseases such as blast and sheath rot, respectively^3,4,6^. Therefore, it is essential to detect the seed-borne inoculum so that an early control measure such as seed treatment, seed certification, and quarantine legislation can be undertaken. Though conventional methods such as visual observations and incubation methods are available, improved methods such as PCR based assays (conventional PCR, qPCR, multiplex PCR, Bio-PCR, nested PCR, etc.) are widely used for routine diagnosis of multiple seed-borne pathogens due to their rapidity, sensitivity, and robustness as compared to the conventional methods^6,8,9,12,26,27^. However, PCR methods require a sophisticated apparatus for thermocycling at different stages of the amplification, which significantly limits more extensive application in under-resourced settings. Therefore, many isothermal amplification methods, such as the LAMP and HDA, which do not require thermocycling, are becoming increasingly popular among the seed pathology laboratories.

The LAMP assay^13^ possesses characteristics such as the non-requirement for a thermocycler, rapid amplification, and high specificity and sensitivity well suited for facilitating point-of-care field applications^19^. Therefore, in the present study, we investigated the applicability of isothermal amplification methods (LAMP and HDA) for sensitive and specific diagnosis of *M. oryzae* and *S. oryzae,* two predominant seed-borne pathogens of rice. The LAMP and HDA assays were optimized for incubation time and template DNA concentration. Both methods were found efficient in detecting the seed-borne *M. oryzae* and *S. oryzae* in seeds. The HDA assay has been widely used to detect viruses and bacteria in the animal system21. Here we report its applicability in the routine diagnosis of the seed-borne pathogens of rice. The Lamp assay results were visualized using EtBr and basic fuchsin dyes, and both were able to distinguish the positive amplification from the healthy control.

Among the different nucleic acid-based techniques tested, the LAMP assay was highly sensitive (up to 100 fg of template DNA) compared to HDA, PCR, and qPCR (Supplementary Fig. 1). Although the HDA assay was less sensitive and required longer assay-time than the LAMP assay, its sensitivity was comparable with qPCR and hence, can be the next option after LAMP assay in the resource-poor settings. The LAMP and HDA assays were highly specific to their target pathogens (*M. oryzae* and *S. oryzae*), as evident from the specificity assays using the template DNA of commonly ice seed-associated fungi such as *B. oryzae, R. oryzae, A. flavus, Penicillium* sp. and *C. fulvum*. Therefore, either the LAMP or HDA assays can be used for diagnosing seed-borne *M. oryzae* and *S. oryzae* pathogens in seeds, and wherein, the LAMP assay was more sensitive and rapid than HDA.

There are many reports where the LAMP assay has been modified into portable devices for better applicability in the resource-poor settings and the point-of-care facilities^23,25^. In this study, we have developed a portable foldable microdevice platform for performing the LAMP assay for the rapid diagnosis of seed-borne *M. oryzae* and *S. oryzae* in rice seeds. This platform was based on the principles developed previously for diagnosing food-borne pathogens^24^. The assay conditions (incubation at 60 °C for 30 min using 100 fg of template DNA) were optimized on the microdevice platform for both *M. oryzae* and *S. oryzae* pathogens. In the newly developed microdevice, the sensitivity, specificity, and assay conditions were similar to conventional LAMP assay. Therefore, this could be used as a prototype for developing on-field diagnostic kits for point-of-care facilities and agriculture extension service persons.

For result visualization, we have tested two colorimetric dyes, and both the dyes showed similar results. However, basic fuchsin was selected for result visualization in the foldable microdevice as it is very safe to use and does not require any sophisticated instruments for result visualization. Previous studies have also reported the feasibility of basic fuchsin dye in the foldable isothermal amplification microdevice for colorimetric detection of multiple food-borne pathogens^24^. The use of basic fuchsin dye also eliminates the limitations of the conventional colorimetric detection methods where pyrophosphate, the end product of LAMP assay, acts as a polymerase inhibitor and interferes with amplification of the target site in the template DNA^28,29^.

The portable, foldable LAMP-based microdevice developed in this study is highly sensitive, specific, cheap, and robust for detecting seed-borne *M. oryzae* and *S. oryzae* inoculum in the rice seeds and, therefore, can be used as a prototype for the development of fabricated diagnostic kits. Early detection of seed-borne pathogens enables taking appropriate precautionary control measures, ensuring high-quality seed standards, and avoiding potential field epidemics.

## Materials and methods

### Source of the fungal culture and rice seeds

Pure cultures of *M. oryzae* strain MG01, *S. oryzae* strain Saro-13, *R. oryzae, A. flavus, Penicillium* sp., and *C. fulvum* were obtained from the Endophytes project, Department of Plant Pathology, University of Agricultural Sciences, Bangalore, India. Pure culture of *B. oryzae* strain Bo-BLR1 (GenBank Accession No.MH481660) was obtained from the Rice Pathology Laboratory, All India Coordinated Rice Improvement Programme, Gangavathi, India. The rice seeds were collected from the blast and sheath rot infected plants (cv. BPT5204), and seeds were collected from the disease-free plants of cv. BPT5204 were served as negative control. Healthy seeds were again confirmed for the absence of seed-borne inoculum through a blotter test as described previously^30^.

### Isolation of fungal genomic DNA from the fungi

The genomic DNA from *M. oryzae* strain MG01, *S. oryzae* strain Saro-13, *R. oryzae, A. flavus, Penicillium* sp., *B. oryzae,* and *C. fulvum* were isolated using the CTAB method^31^. RNA contamination was removed by digesting the obtained genomic DNA sample with 10 µg/mL RNase. The isolated genomic DNA was quantified using Nanodrop (Model-DS-11 FX + , DeNovix, USA).

### Genomic DNA isolation from rice seeds

Genomic DNA was isolated from rice seeds by the CTAB method. RNA contamination was removed by digesting the obtained genomic DNA sample with 10 µg/mL RNase. Similarly, DNA from the rice seeds was also extracted using a high-throughput DNA extraction method^32^. The extracted DNA quality was assessed using Nanodrop (Model-DS-11 FX + , DeNovix, USA).

### Primer design

Complete details of the different primers designed have been described in Table 1. The PCR and HDA primers were designed using the Primer3.4.1 program (http://primer3.ut.ee). For qPCR, forward and reverse primers were designed using IDT analyzer software (https://www.idtdna.com). Four sets of loop primers for the LAMP assay were designed using Primer Explorer V5 (https://primerexplorer.jp/e/). The loop primers included two inner primers, a forward inner primer (FIP) and a backward inner primer (BIP), and two outer primers F3 and B3. FIP consisted of F1c and F2 regions, and BIP consisted of B1c and B2 regions (Supplementary Fig. 2 and 3).

**Table 1:** Oligonucleotide primers used for different assays.

For the PCR, HDA, and qPCR assays					
Pathogen	Type	Primer sequence	Primer coordinates	Reference ID	Target gene
*M. oryzae*	Forward	AGGCTCACCACATTCTCAAG	521–539	XM_003713594	RNA *polymerase* II largest subunit
	Reverse	GTCGCCAAGCTTGTACGT	699–716		
*S. oryzae*	Forward	CGACAAGAAGAACCCTCGC	35–53	MW314046	RNA *polymerase* II largest subunit
	Reverse	CAGGATCTCTTTCATCCGCC	348–368		
**For the LAMP assay**					
*M. oryzae*	F3	AGGCTCACCACATTCTCAAG	521–539	XM_003713594	RNA *polymerase* II largest subunit
	B3	GTCGCCAAGCTTGTACGT	699–716		
	FIP	GCCATTCGGGTCTGGCATAGTC-TATTCCCGAAGACGACCTGT	FIP (F1c + F2):581–603 + 542–561		
	BIP	CGTCACGGTTCTGCCAGTGC-TACGCATTCCCTGCGATG	BIP (B1c + B2):608–627 + 667–684		
	LF	CTTGTTCAGGCCCATCTTCC	562–581		
	LB	CCTCCCGTCAGGCCCAGTAT	633–652		
*S. oryzae*	F3	CGACAAGAAGAACCCTCGC	27,193–27,211	MW314046	RNA *polymerase* II large subunit
	B3	CAGGATCTCTTTCATCCGCC	27,406–27,425		
	FIP	TAGCTTGCCCACATGTTCTGCA-GAAGGACTCAACGATCCGAG	FIP (F1c + F2) (106–128 + 55–77)		
	BIP	TTGCCTGCCCAGTCTATCACC-AGCAGTTGTGGCAAACAATC	BIP (B1c + B2) (264–284 + 319–338)		
	LF	TACTGCCTGTCAATAGAGCC	82–101		
	LB	GCTACATCAAGAAAGTCAA	288–306		

### The PCR assay

The PCR assay was conducted in a 10 µl reaction mixture containing 5 µl of 2 × master mix (Takara, Japan), 3 µl of nuclease-free water, 10 pmol/µleach of forward and reverse primers, 50 ng of template DNA. The assay was performed in a thermal cycler (Eppendorf-vapo. protect, Germany). PCR conditions were as follows: 95 °C for 5 min, 30 cycles of denaturation (95 °C for 30 s), annealing (56 °C for 30 s), and extension (72 °C for 30 s), followed by a final extension at 72 °C for 10 min.

### The qPCR assay

The assay was performed as described previously^33,34^. Briefly, the reaction mixture contained 10 μl of SYBR Green I, 1 μl of each (2 pmol/µl) forward and reverse primers along with 50 ng of template DNA, and finally, nuclease-free water was used to make up the final volume. The amplification conditions for the reaction was 95 °C for 30 s, with 35 cycles of 95 °C for 5 s, followed by 56 °C for 30 s, 72 °C for 20 s, and fluorescence read at 72 °C at the end of each cycle, with a final melting curve at 65 °C to 95 °C. The assay results were analyzed by plotting the log of the template DNA concentration against cycle threshold (*C*_t_) values.

### The HDA assay

The HDA assay was performed using the IsoAmp II universal tHDA Kit (BioLab Inc., New England Biologicals, USA). The reactions were carried out in 50 μL total volume described previously by Barreda-García et al. (2016 and 2018)^21,35^. In the assay, two separate reaction mixtures, A and B, were prepared. Mix A was incubated at 95 °C for 2 min and immediately transferred into ice for another 5 min. Mix B (25 μL), containing 10 μL of nuclease-free water, 2.5 μL of 10 × annealing buffer, 1.5 μL of 100 mM MgSO_4_, 4 μL of 500 mM NaCl, 3.5 μL of IsoAmpdNTP solution, and 3.5 μL of IsoAmp enzymes mixture, was then added and the new mixture was incubated in a water bath at 56 °C for 60 min.

### The LAMP assay

The LAMP assay was carried out in a 25 µl reaction mixture containing 1 × Thermo pol buffer pH 8.8 (10 mM (NH4)_2_SO_4_, 20 mM Tris–HCl, 10 mM KCl, 2 mM MgSO_4_, 0.1% Triton-X), 0.2 M betaine, 0.6 mM of dNTPs, 10 pmol of F3/B3 primers, 20 pmol of FIP/BIP primers, 8 U of *Bst* polymerase large fragment (New England Biologicals, USA), template DNA with a concentration of 100 ng/µl and the reaction volume was made up to 25 µl using sterile distilled water (Supplementary Table 2). The LAMP reaction mixture was optimized for reaction temperature (from 50 to 66 °C) and reaction time (from 10 to 90 min). The optimized time and temperature were further used to assess the LAMP assay's sensitivity at different template DNA dilutions (10 ng to 100 fg). The reaction was also terminated by heat inactivation at 80 °C for 10 min in a water bath (KEMI, India). The assay results were visualized using ethidium bromide (EtBr) and basic fuchsin dyes. About 1 µl EtBr dye (10 mg/mL) was added post-reaction, whereas 1 µl of basic fuchsin (9 μM) was added post the reaction (Supplementary Table 3). The products were visualized directly after the completion of the process. All reactions were performed in triplicates.

### The specificity of the LAMP assay

The LAMP assay's specificity for detecting *M. oryzae* and *S. oryzae* was evaluated using template DNA of other rice-seed associated fungi such as *B. oryzae, R. oryzae, A. flavus, Penicillium* sp., and *C. fulvum*.

### The sensitivity of different nucleic acid amplification techniques

The sensitivity of each nucleic acid amplification technique (PCR, qPCR, HDA, and LAMP) was tested at different DNA template concentrations viz., 10 ng, 1 ng, 100 pg, 50 pg, 10 pg, 100 fg, and 50 fg. Pathogen-specific (*M. oryzae* and *S. oryzae*) primers of PCR, qPCR, HDA, and LAMP assays were tested against different template concentrations using optimized amplification conditions.

### Foldable membrane microdevice platform

**Figure 7:**
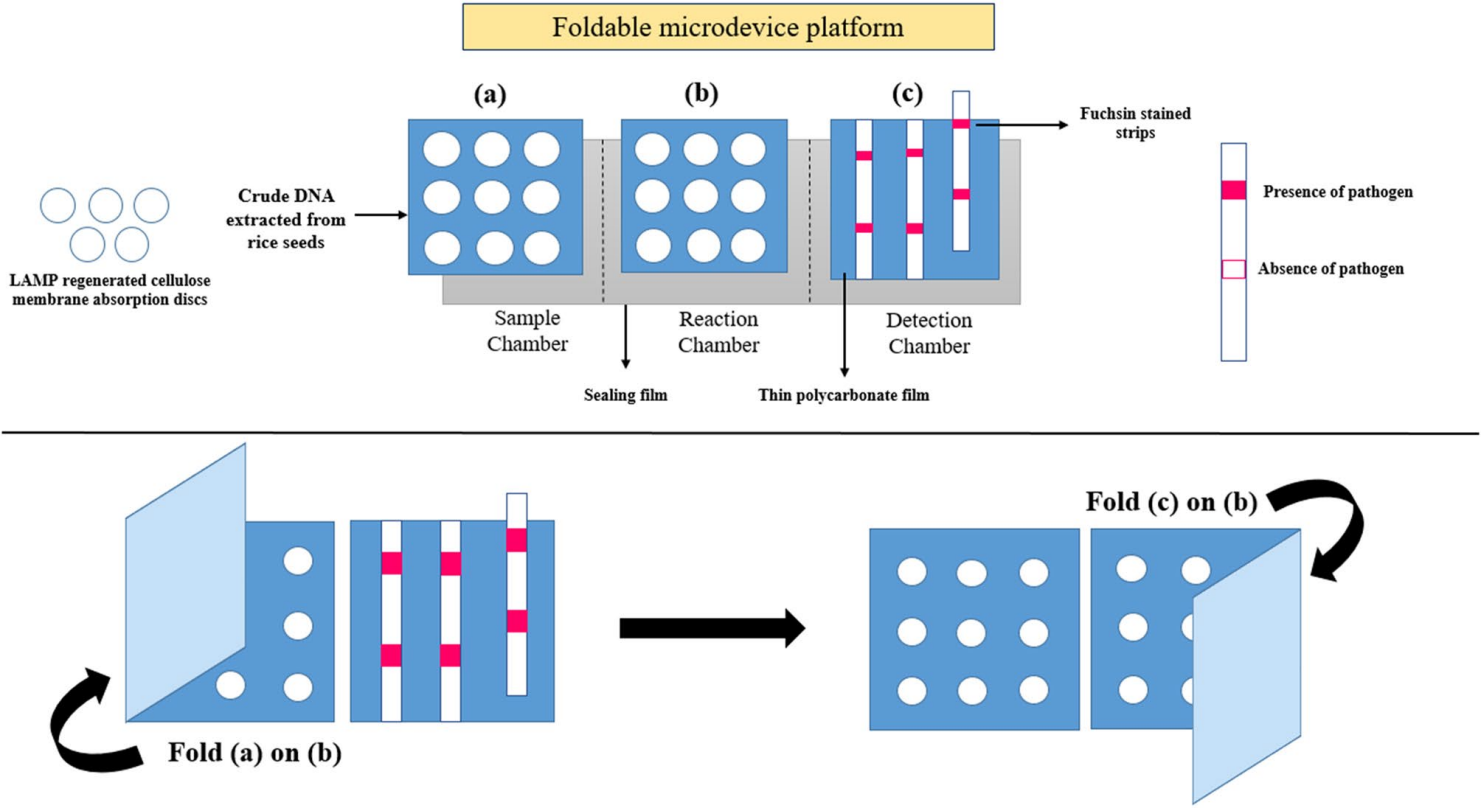
Overview of the membrane-based microdevice design. (**a**) Sample chamber with nine absorption discs, (**b**) reaction chamber, and (**c**) detection chamber with fuchsin stained strips.

A PCR sealing film (40 × 90 mm) was used as a bottom layer to develop a portable, foldable membrane-based microdevice for performing the LAMP assay. The PCR film was divided into three chambers viz., sample chamber, reaction chamber, and a detection chamber as reported previously^24^. The reaction chamber was covered with polycarbonate (PC) film (40 × 30 mm), and nine reaction wells (2.5 mm radius each) were cut out of the PC film. The detection chamber was coated with basic fuchsin dye for result visualization. The basic step involved in designing a membrane microdevice is illustrated in Fig. 7. In the sample chamber, 10μL of template DNA (≈100 fg) was added and allowed for 2 min at room temperature.

Similarly, in the reaction chamber, 10μL of LAMP reaction mixture (buffer, enzyme, and primers) was added to each reaction well and allowed for 2 min at room temperature. Later, the sample chamber was folded onto the reaction chamber and was finger pressed firmly for a min and then separated. The reaction chamber was then covered with a sealing film to avoid the reagents evaporation and incubated at 60 °C for 30 min. After incubation, the sealing film was removed, and about 1μL of HCl was added to each reaction wells, and again incubated at 58 °C for 5 min. To the incubated reaction chamber, 2 μL of Sodium sulfite was added and folded onto the detection chamber to visualize the result. The results were inferred based on the color change either from magenta to colorless (for negative amplification) or magenta to purple (for positive amplification). This method was standardized separately for the detection of *M. oryzae* and *S. oryzae*.

### Statistical analysis

Statistical analysis was carried out using two-way factorial analysis and a completely randomized design. The ANOVA for the variance between the groups, namely time, temperature, and template, were analyzed in R studio (Version 1.1.) using the package ggpurb and ggplot2 (Supplementary Fig. 4).

## Supplementary Information

Supplementary Information 1.
