# Supplementary material for: LAMP-based foldable microdevice platform for the rapid detection of Magnaporthe oryzae and Sarocladium oryzae in rice seed
Source: Sci Rep. 2021 Jan 8;11:178. doi: 10.1038/s41598-020-80644-z (PMC7794292; doi:10.1038/s41598-020-80644-z)
Supplement: Supplementary file 1 — Supplementary Information 1. [file 41598_2020_80644_MOESM1_ESM.docx]

**LAMP-based foldable microdevice platform for the rapid detection of *Magnaporthe oryzae* and *Sarocladium oryzae* in rice seed**

M. K. Prasannakumar^1^*, P. Buela Parivallal^1^, Devanna Pramesh^2^*, H.B. Mahesh^3^ and Edwin Raj^4^

^1^Department of Plant Pathology, University of Agricultural Sciences, Bangalore, India.

^2^Rice Pathology Laboratory, All India Coordinated Rice Improvement Programme, University of Agricultural Sciences, Raichur, India.

^3^Department of Genetics and Plant Breeding, College of Agriculture, V.C. Farm, Mandya, India.

^4^Plant Pathology Division, ICAR - National Research Center for Banana, Thayanur, India.

*Corresponding authors:

E.mail: [babu_prasanna@rediffmail.com](mailto:babu_prasanna@rediffmail.com) (MKP); [pramesh84@uasraichur.edu.in](mailto:pramesh84@uasraichur.edu.in) (DP)

**
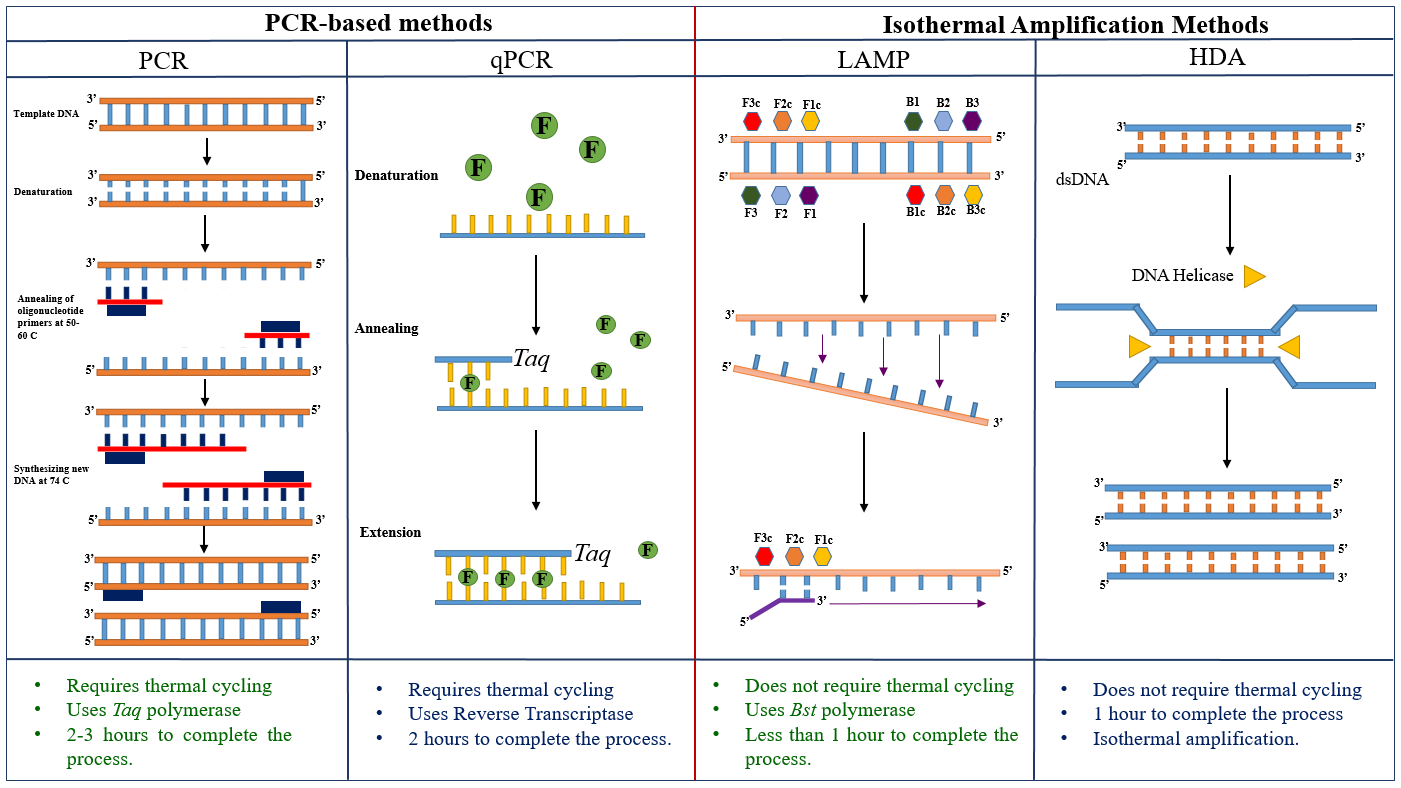
**

**Supplementary figure S1.** Graphical abstract

**
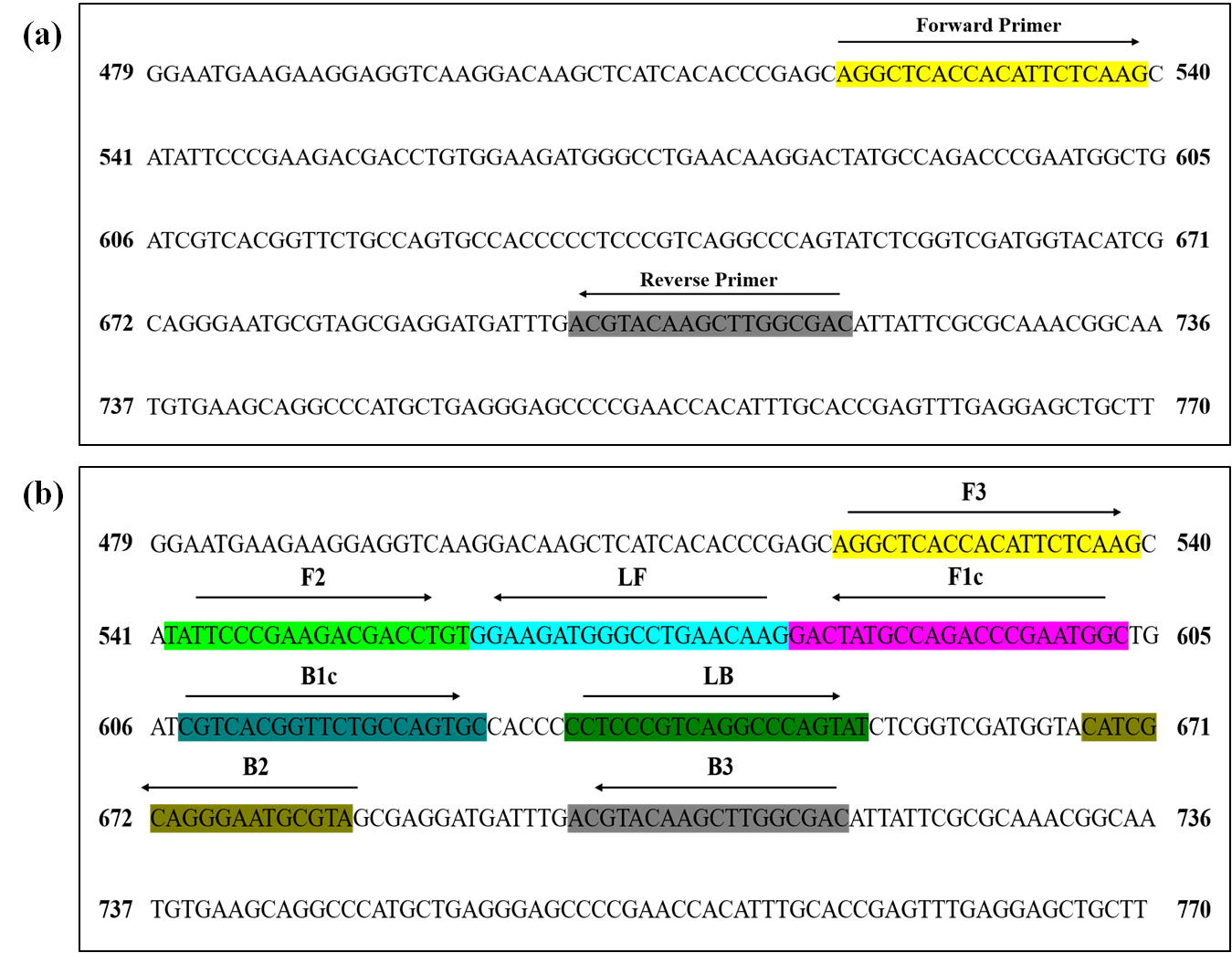
**

**Supplementary figure S2.** The primer sequences and their coordinates designed explicitly for detecting *Magnaporthe oryzae* in the PCR, HDA, and qPCR assays (a); in the LAMP assay (b). Color coding indicates the primers coordinates, and arrows indicating the direction of extension.

**Supplementary figure S3.** The primer sequences and their coordinates designed explicitly for detecting *Sarocladium oryzae* in the PCR, HDA, and qPCR assays (a); in the LAMP assay (b). Color coding indicates the primers coordinates, and arrows indicating the direction of extension.

**
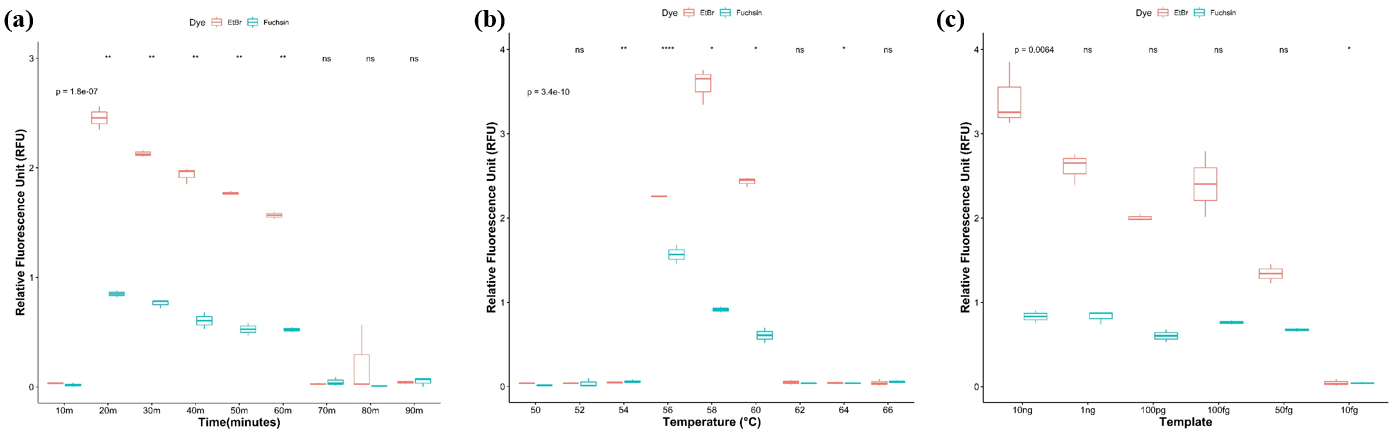
**

**Supplementary figure S4.** Statistical analysis. a) **Time optimization for the LAMP assay**. Box plot represents the relative fluorescence unit (RFU) of different dyes (Ethidium bromide (EtBr) and Basic Fuchsin). The lower boundary of the box indicates the standard deviation. The *p* values in the graph using the variance of Anova among the groups (Time) revealed 95% confidence at *p* is ≤ 0.05, and 99% confidence at *p* is ≤ 0.01. At the same time, the t-test between the treatments showed significance ≤ 0.05 (*) and ≤ 0.01 (**). b) **Temperature optimization for the LAMP assay**. Box plot representing the relative fluorescence unit (RFU) of different dyes (Ethidium bromide (EtBr) and Basic Fuchsin), the lower boundary of the box indicates the standard deviation. The *p* values in the graph using the variance of Anova among the groups (Temperatures) revealed 95% confidence at *p* is ≤ 0.05, and 99% confidence at *p* is ≤ 0.01. At the same time, the t-test between the treatments showed significance ≤ 0.05 (*) and ≤ 0.01 (**). c) **Template concentration optimization for the LAMP assay**. Box plot representing the positive detection describing median (thin black line), the lower boundary of the box indicates the standard deviation. The *p* values in the graph using the variance of Anova among the groups (Template concentration) revealed 95% confidence at *p* is ≤ 0.05, and 99% confidence at *p* is ≤ 0.01. At the same time, the t-test between the treatments showed significance ≤ 0.05 (*) and ≤ 0.01 (**).

**Supplementary Table 1.** The specificity of the LAMP assay for the detection of *Magnaporthe oryzae* and *Sarocladium oryzae* genes

| Pathogens | The LAMP-specific primers | |
| --- | --- | --- |
|  | *Magnaporthe oryzae* | *Sarocladium oryzae* |
| *Magnaporthe oryzae* | + | - |
| *Sarocladium oryzae* | - | + |
| *Bipolaris oryzae* | - | - |
| *Rhizopus oryzae* | - | - |
| *Aspergillus flavus* | - | - |
| *Penicillium* | - | - |
| *Cladosporium*  *fulvum* | - | - |

**Supplementary Table 2.** Reaction setup of the LAMP assay used in this study

| Sl. No | Components | Stock concentration | Working concentration | Required volume per reaction (μL) |
| --- | --- | --- | --- | --- |
| 1 | 10X Thermopol Buffer | 10 X | 1 X | 2.5 |
| 2 | dNTP’s | 10 mM | 0.6 mM | 1.5 |
| 3 | Betaine | 5 M | 0.2 M | 1.0 |
| 4 | F3 | 100 pM | 10 pM | 2.0 |
| 5 | B3 | 100 pM | 10 pM | 2.0 |
| 6 | FIP | 100 pM | 10 pM | 2.0 |
| 7 | BIP | 100 pM | 10 pM | 2.0 |
| 8 | LF | 100 pM | 10 pM | 2.0 |
| 9 | LB | 100 pM | 10 pM | 2.0 |
| 10 | *Bst polymerase* | 8 Units | 8 Units/ μL | 1.0 |
| 11 | Nuclease free water | - | - | Variable |
| 12 | Template DNA (50ng/μL) | - | - | Variable |
|  | Total Volume | 25.0 | | |

**Supplementary Table 3.** Details of the DNA binding dyes used in this study

|  | Ethidium bromide | Basic Fuchsin |
| --- | --- | --- |
| Stock concentration | 10mg/ mL | 9 μM |
| Volume per μL | 1.0 | 1.0 |
| Detection | UV Transilluminator | Direct Visualisation |
| Field applicability | - | + |
